# Young people’s contribution to the *Global strategy for women’s, children’s and adolescents’ health (2016–2030)*

**DOI:** 10.2471/BLT.16.174714

**Published:** 2016-05-02

**Authors:** Zanele Mabaso, Temitayo Erogbogbo, Kadidiatou Toure

**Affiliations:** a Girls’ Globe, Pretoria, South Africa.; b The Partnership for Maternal, Newborn & Child Health, World Health Organization, avenue Appia 20, 1211 Geneva 27, Switzerland.

The world has 1.8 billion people aged 10–24 years.^1^ Despite being the healthiest group of the general population, 1.3 million adolescents (aged 15–24 years) die annually from preventable causes.^2^ One in 10 girls under the age of 20 has been a victim of sexual violence.^3^ Fifteen million girls under the age of 18 get married every year.^4^ Over 500 million people aged 15–24 years live on less than 2 United States dollars (US$) a day.^5^ However, there is now an increased recognition of the need to invest in young people. The behaviours shaped in adolescence can have long term effects.^6^ Investing in adolescent health and well-being is therefore central to the achievement of human rights and equity, as well as improving social and economic development.^7^^,^^8^

The sustainable development goals (SDGs) and the *Global strategy for women’s, children’s and adolescents’ health (2016–2030)*^7^ prioritize young people.^7^^,^^9^ Efforts to meaningfully engage young people have also increased; this approach was developed with the active participation of young people and it focuses on adolescents and their distinct health needs.^2^ Participants to the development of the strategy called for inclusion of the leading causes of morbidity and mortality for young people – such as road traffic injuries and noncommunicable diseases. They advocated for a multisectoral approach to addressing the social, cultural, economic and legal barriers often faced by young people and for identification of data gaps.^10^^,^^11^

Young people are supporting the implementation of the global strategy. In October 2015, the Partnership for Maternal, Newborn & Child Health created an adolescent and youth constituency that represents youth-led organizations. Their representation is meant to integrate young people’s perspectives in the partnership’s activities supporting the global strategy. The constituency’s objectives for 2016 include: strengthening the role of young people in accountability, advocating for comprehensive national adolescent health policies and supporting the implementation of the global strategy. Young people’s priorities are also featured in supportive mechanisms such as the global financing facility and the unified accountability framework.

Meaningfully engaging young people is an important component in achieving the targets of the global strategy. Stakeholders should engage young people to define and deliver policies that shape their future.
